# Eosinophilic Esophagitis for the Otolaryngologist

**DOI:** 10.1155/2012/181402

**Published:** 2011-12-15

**Authors:** Petros D. Karkos, R. Srivastava, S. Kaptanis, C. Vaughan

**Affiliations:** Department of Otolaryngology, Bradford Royal Infirmary, Bradford BD96RJ, UK

## Abstract

*Objectives*. This paper focuses on current diagnostic and treatment options for Eosinophilic Esophagitis (EE). *Study Design*. literature review. *Results*. EE can be suspected on history and endoscopy although definitive diagnosis is strictly based on histopathology. It is a relatively new entity and is often misdiagnosed as gastroesophageal reflux (GERD). Eosinophilic infiltration of the esophageal mucosa is responsible for esophageal symptoms which can range from mild to debilitating dysphagia and food impaction, when untreated. In fact recurrent foreign body and food impaction can often be blamed for undiagnosed EE. There seems to be a strong familial component and association with allergy. The introduction of transnasal esophagoscopy in adult laryngology has enabled otolaryngologists to readily diagnose EE and promoted awareness of this often difficult to recognize entity. *Conclusions*. Despite higher awareness, the literature suggests that EE remains a commonly misdiagnosed condition especially in the otolaryngology community. Genetic studies are required to unfold the true familial and genetic component of this fascinating entity.

## 1. Introduction

Eosinophilic esophagitis (EE) previously known as idiopathic eosinophilic oesophagitis, atopic oesophagitis, and allergic oesophagitis is a clinicopathological entity that is being diagnosed with increasing frequency. According to the latest consensus EE represents a chronic immune/antigen-mediated esophageal disease characterized clinically by symptoms related to esophageal dysfunction and histologically by eosinophil-predominant inflammation [1]. This disease is isolated to the esophagus and has to be distinguished of any moderate eosinophilic infiltration associated to a generalized eosinophilic infiltration of a gut mucosa (gastroenteritis and colitis) [1]. The diagnostic criteria of EE include esophageal and/or upper gastrointestinal tract symptoms accompanied by ≥15 intraepithelial eosinophils/high power field (HPF) in 1 or more biopsy specimens without pathologic gastroesophageal reflux disease (GERD), as shown by normal pH monitoring of the distal esophagus or the lack of response to high-dose proton pump inhibitor (PPI) medication [2].

## 2. Epidemiology

EE was thought to be a rare condition; however a sharp rise in its prevalence is recognized in most countries. This could be due to a combination of a true escalation in its incidence, combined with an increasing recognition, awareness, and testing amongst gastroenterologists, otolaryngologists, and pathologists. This notion is supported by the fact that there are numerous reports of patients with multiple oesophageal rings with intraepithelial eosinophils that had been ascribed to acid reflux, but who did not respond to standard acid suppression therapy. In retrospect, these patients may have had EE [3–5].

 The literature reveals an increase in frequency in both pediatric [6] and adult [7] populations. One particular north American study showed that the incidence of EE has increased dramatically from 0.35 per 100 000 between 1991 and 1995 to 9.45 per 100000 between 2001 and 2005 making the prevalence of EE 55.0 per 100,000 people [8].

EE affects both sexes and all age groups with the typical patient being an atopic male presenting in childhood or the 3rd or 4th decades of life [1]. The age at diagnosis can vary though. The disease affects 8 children and one adult, and most pediatric cases appear in the first three years [1, 3]. Moreover, children with eosinophilic esophagitis have a higher frequency of atopic symptoms and peripheral eosinophilia than do adults [7]. The male-to-female prevalence ratio has been reported as 3 : 1 with cases extensively reported in patients of different ethnic origins [3]. Familial trends have been reported [9–12] with the majority of cases to date reported from North America and Europe and to a lesser extent Asia, Australia, and South America. No cases have been reported from Africa [10].

## 3. Etiology

There are a number of factors that are believed to play a role in the origin of EE. These are genetics, allergy, seasonal variation, and GERD.

 There is more literature to support a genetic basis for EE. Studies have validated the expression of a unique EE transcriptome and validated that it differentiates EE from GERD, with eotaxin-3 being abundantly overexpressed in patients with EE [1, 13]. IL-13 has been found to be specifically upregulated in the esophagi of patients with EE and might function as a master regulator of the EE transcriptome [14]. Rothenberg et al. have identified the first genome-wide susceptibility locus at 5q22 [15]. Sherrill et al. have reported that polymorphisms in the thymic stromal lymphopoietin (TSLP) gene are risk factors for EE independent of underlying allergy phenotypes [16]. They state there is a gender-specific association between single-nucleotide polymorphisms (SNPs) in TSLP as well as a nonsynonymous SNP in the TSLP receptor which suggests a mechanism for the male predilection of the EoE [16]. Another SNP in the promoter of the TGF-*β*1 gene has been linked to reduced esophageal remodeling following topical steroid treatment. Familiar cases have also been reported [17].

More studies are supporting the concept that EE is an antigen-driven allergic condition, with a varying percentage of pediatric and adult patients having at least one more “allergic” disease. It is reported that 50%–60% of patients with EE have a prior history of atopy [1, 8, 18]. The majority of patients have evidence of or a familiar history of allergic rhinitis, asthma, eczema, or hypersensitivity to foods or aeroallergens. The latter two are based on skin prick testing and IgE test results. Moawad et al. demonstrated a seasonal variation in the diagnosis of EE which correlated with higher pollen count [19]. However, EE was still present during periods of lower atmospheric pollen concentrations and in patients without a history of atopic disease, pointing towards a possible multifactorial pathogenesis. In the pediatric literature, food allergies have been implicated in the pathogenesis of EE [19, 20].

 Allergens induce T-helper-2 (Th2) cells to produce interleukin (IL)-13, which can cause esophageal cells to over-express eotaxin-3 and fibroblasts. Activated Th2 cells also produce IL-5, which regulates eosinophil numbers and their response to eotaxin-3. In addition to eosinophils, mast cells and lymphocytes (including B cells) accumulate in the esophagus to contribute to the local inflammatory responses observed in patients with EE. The resulting injury leads to esophageal remodelling with wall thickening and fibrosis [9]. The cytotoxic role of eosinophils in EE is directly related to the observed histopathological changes with destruction of the most superficial epithelial layers and the regenerative response from the basal layers of the epithelium [21, 22].

 The exact role of GERD in the development of EE is unclear; however the latest updated consensus recommendations states esophageal pH monitoring is useful to establish whether GERD is present in EE or not [1].

## 4. Clinical Presentation

Clinical manifestations in children are less specific, whereas in adults they are more predictable. Feeding difficulties are the manifestation in infants and toddlers, whereas vomiting and/or pain may be present in school-aged children. Dysphagia is the main symptom in adolescents [1, 5]. Other symptoms include a failure to thrive, heartburn, and isolated nausea [9]. To date, no pathognomonic features have been identified. Atopy is a common association in pediatric and adult patients with evidence of another allergic disease (allergic rhinitis, asthma, eczema, or hypersensitivity to foods or aeroallergens) in more than half the cases. Family history of atopy is frequent, with one study reporting a rate of 74% [9, 23].

 The most common presenting symptom in adults is solid food dysphagia. Others include food impaction (which may or may not require endoscopic intervention), chest and upper abdominal discomfort/pain, and resistant reflux symptoms (despite a trial of acid suppression). One study showed that more than half of patients with esophageal food impaction, based on clinicopathologic features are likely to have EE [24].

 There is a subset of patients who have symptoms of EE, have had GERD diagnostically excluded but still demonstrate a clinicopathologic response to PPIs. Terms used to describe these patients include PPI-responsive esophageal eosinophilia. The definition and diagnostic guidelines of EE include the term immune/antigen driven; however, studies and clinical experience have identified a potential anti-inflammatory or “barrier healing” role for proton pump inhibition in patients with esophageal eosinophilia [1].

## 5. Diagnosis

Esophagoscopy with biopsy is the ideal investigation for the diagnosis of EE. There are various endoscopic findings, however none pathognomonic for EE. These include mucosal fragility (59% of cases), esophageal “trachealization” (multiple concentric rings resembling the trachea) in 49%, strictures in 40% of cases, furrows, white plaques, or papules in 16% (aggregates of eosinophilic microabscesses), irregular mucosa, reddish changes in esophageal mucosal pattern, esophageal tears, and a narrow caliber in 5% [10]. Many of these features, including longitudinal furrows, are subtle and can be missed. Between 9% and 32% of patients with symptoms suggesting eosinophilic esophagitis have normal endoscopic findings, and studies have shown patients can have histologically proven EE yet normal macroscopic appearance on endoscopy [25].

 Radiological investigations are not recommended except in selected cases in order to elicit anatomical abnormalities/variations.

 Histology is essential in making the diagnosis of EE. The latest consensus recommends that 2 to 4 mucosal biopsy specimens of the proximal and distal esophagus should be obtained, as various studies have shown the eosinophilic infiltration is similar in these sections of the esophagus. In children and, when indicated in adults, biopsy specimens of the gastric antrum and duodenum should be obtained once to exclude other potential causes of esophageal eosinophilia [1]. No prospective studies have determined a threshold number of esophageal eosinophils that can establish a diagnosis of EE with high specificity and sensitivity and consistently allow differentiation of EE from other causes of esophageal eosinophilia. It is recommended that, until more studies are performed, all histologic features including eosinophil microabscess formation, superficial layering of eosinophils, extracellular eosinophil granules, basal cell hyperplasia, dilated intercellular spaces, rete peg elongation, subepithelial lamina propria fibrosis, and increases in other cell types be noted in pathology reports [1].

 One-third to one-half of patients have peripheral eosinophilia, and up to 55% have increased serum levels of immunoglobulin E (IgE), therefore the search for specific IgEs is strongly advised [20]. Although peripheral eosinophilia can correlate with tissue eosinophilia in some patients with EE, changes in the former need to be considered with caution [1]. Because of the association with allergic diseases, a complete evaluation of aeroallergen and immediate type food allergy is warranted.

## 6. Treatment

Management of patients EE remains controversial. EE is a chronic disease and its activity may fluctuate independently of any therapeutic intervention, and, although it affects quality of life, it does not seem to limit life expectancy or be associated with malignant or premalignant conditions. Several treatment modalities have been tested. We focus on medical, dietary, and surgical interventions according to the 2011 recommendation [1], which have recently replaced the 2007 recommendations.

Surgical intervention is usually reserved for the complications of EE, namely, stenosis of the esophagus. Esophageal dilation with either Savary dilators or unsedated transnasal balloon techniques is associated with an 83% symptom response rate and a low complication rate of 5% [26]. Other smaller studies report excellent symptomatic relief, for both adults and children [27, 28]. Although dilation does not improve the underlying inflammatory process and will probably need to be repeated, perhaps it is an adequate strategy for the healthy, young to middle-aged men commonly affected by the disease, who might prefer it to regular medications or diet [29]. Although complications are more frequent than those associated with dilation for other benign conditions [30], the risk of perforation appears to have been exaggerated—a systematic review of 18 studies [31] identified 1 perforation in 671 dilations (0.1% risk).

Medical treatment for EE needs to be both effective and safe, considering the fact that EE is a chronic disease requiring prolonged courses of therapy for remission. Medications used include corticosteroids, cromolyn sodium, proton-pump inhibitors (PPIs), leukotriene receptor antagonists, immunosuppressive agents, and monoclonal antibodies.

PPIs are used both for diagnostic and therapeutic purposes. Acid suppression is important to rule out secondary esophageal eosinophilia due to GERD, although some authors feel EE's contribution to refractory GERD is not significant [32]. A high PPI dose of 20–40 mg twice daily is recommended for 8 to 12 weeks in adults (1 mg/kg twice daily in children with the adult dose as maximum). PPI therapy alone is not effective for patients with EE; however it might alleviate symptoms related to secondary GERD [1]. It has been suggested that PPI-responsive esophageal eosinophilia is a different clinical entity. A small randomized controlled trial comparing PPIs versus topical corticosteroids failed to show a difference between the groups [33].

Topical corticosteroids are effective in children and adults, inducing remission in most cases. A number of randomized controlled trials have evaluated this in recent years: a 15-day course of treatment with budesonide (viscous suspension, 1 mg twice daily for adults, 0.5 mg twice daily for children under 10 years old) is well tolerated and highly effective in inducing a histologic and clinical remission [1]. Fluticasone (440–880 mg twice daily for adults, 88–440 mg twice to 4 times daily for children, puffed and swallowed through a metered-dose inhaler) has also been used in adults and children and was favoured before 2007 [33]. There is no significant evidence that either treatment is superior to the other [1]. Systemic corticoids are not recommended due to their adverse effects (up to 40%) as other treatments are almost equally effective [33]. For severe urgent cases requiring hospitalization, Prednisone 1-2 mg/kg should be considered [1].

Other treatment modalities [1, 33] include

- cromolyn sodium, which was shown to be ineffective and is not recommended,
- leukotriene receptor antagonists, of which montelukast might have a role in maintaining remission in children but was proven inefficient in adults and is not recommended,
- immune suppressants, such as azathioprine or 6-mercaptopurine, which were shown to be ineffective and is not recommended
- monoclonal antibodies, such as Mepolizumab, a humanized monoclonal antibody against interleukin-5 (IL-5) which is not effective in adults and although it promotes a histologic response it is not clinically effective in children, Omalizumab (a monoclonal anti-IgE antibody) which is not effective [34], and Infliximab (a chimeric monoclonal anti tumor necrosis factor-*α* antibody) which is not effective.

Diet modification (elimination of specific foods guided by skin prick and atopy patch testing) is effective in over 75% of patients and should be attempted in children. In adults results are mixed, possibly because of poor compliance [1]. Elemental diet is not a real option in adults and elimination diets (directed, or empirical of milk, egg, soy, peanut, tree nut, wheat, shellfish, and fish) do not achieve consistent results.

## 7. Conclusions

Despite higher awareness, the literature suggests that EE remains a commonly misdiagnosed condition especially in the otolaryngology community. The introduction of Transnasal Esophagoscopy in the ENT office over the last decade has meant that increasingly more and more laryngologists become accustomed in recognising esophageal pathology including EE.

Genetic studies are required to unfold the true familial and genetic component of this fascinating entity.
